# Multi Frequency Phase Fluorimetry (MFPF) for Oxygen Partial Pressure Measurement: *Ex Vivo* Validation by Polarographic Clark-Type Electrode

**DOI:** 10.1371/journal.pone.0060591

**Published:** 2013-04-02

**Authors:** Stefan Boehme, Bastian Duenges, Klaus U. Klein, Volker Hartwich, Beate Mayr, Jolanda Consiglio, James E. Baumgardner, Klaus Markstaller, Reto Basciani, Andreas Vogt

**Affiliations:** 1 Department of Anaesthesia, General Intensive Care and Pain Management, Medical University of Vienna, Vienna, Austria; 2 Department of Anesthesiology, Medical Center of the Johannes Gutenberg-University, Mainz, Germany; 3 Oscillogy, Folsom, Pennsylvania, United States of America; 4 Department of Anesthesiology and Pain Therapy, Inselspital, Bern University Hospital, and University of Bern, Bern, Switzerland; 5 Department of Cardiovascular Surgery, Swiss Cardiovascular Center, Bern University Hospital, Bern, Switzerland; UC Davis School of Medicine, United States of America

## Abstract

**Background:**

Measurement of partial pressure of oxygen (P_O2_) at high temporal resolution remains a technological challenge. This study introduces a novel P_O2_ sensing technology based on Multi-Frequency Phase Fluorimetry (MFPF). The aim was to validate MFPF against polarographic Clark-type electrode (CTE) P_O2_ measurements.

**Methodology/Principal Findings:**

MFPF technology was first investigated in N = 8 anaesthetised pigs at F_IO2_ of 0.21, 0.4, 0.6, 0.8 and 1.0. At each F_IO2_ level, blood samples were withdrawn and P_O2_ was measured *in vitro* with MFPF using two FOXY-AL300 probes immediately followed by CTE measurement. Secondly, MFPF-P_O2_ readings were compared to CTE in an artificial circulatory setup (human packed red blood cells, haematocrit of 30%). The impacts of temperature (20, 30, 40°C) and blood flow (0.8, 1.6, 2.4, 3.2, 4.0 L min^−1^) on MFPF-P_O2_ measurements were assessed. MFPF response time in the gas- and blood-phase was determined. Porcine MFPF-P_O2_ ranged from 63 to 749 mmHg; the corresponding CTE samples from 43 to 712 mmHg. Linear regression: CTE = 15.59+1.18*MFPF (R^2^ = 0.93; *P*<0.0001). Bland Altman analysis: mean_diff_ 69.2 mmHg, range_diff_ -50.1/215.6 mmHg, 1.96-SD limits -56.3/194.8 mmHg. In artificial circulatory setup, MFPF-P_O2_ ranged from 20 to 567 mmHg and CTE samples from 11 to 575 mmHg. Linear regression: CTE = −8.73+1.05*MFPF (R^2^ = 0.99; *P*<0.0001). Bland-Altman analysis: mean_diff_ 6.6 mmHg, range_diff_ -9.7/20.5 mmHg, 1.96-SD limits -12.7/25.8 mmHg. Differences between MFPF and CTE-P_O2_ due to variations of temperature were less than 6 mmHg (range 0–140 mmHg) and less than 35 mmHg (range 140–750 mmHg); differences due to variations in blood flow were less than 15 mmHg (all *P*-values>0.05). MFPF response-time (monoexponential) was 1.48±0.26 s for the gas-phase and 1.51±0.20 s for the blood-phase.

**Conclusions/Significance:**

MFPF-derived P_O2_ readings were reproducible and showed excellent correlation and good agreement with Clark-type electrode-based P_O2_ measurements. There was no relevant impact of temperature and blood flow upon MFPF-P_O2_ measurements. The response time of the MFPF FOXY-AL300 probe was adequate for real-time sensing in the blood phase.

## Introduction

The polarographic Clark-type electrode remains the gold standard for oxygen partial pressure (P_O2_) measurement to date, and is the underlying electrochemical principle of conventional blood gas P_O2_ analysis [1], [2], [3], [4]. Moreover, polarographic P_O2_ probes, such as the Licox probe, are used for the continuous monitoring of oxygen. These probes provide favourable properties, including high accuracy and stability; however, they lack high temporal resolution [5]. Several publications reveal that oxygen levels may largely fluctuate over time in the systemic (e.g., thoracic aorta) or capillary circulation (e.g. vasomotion, brain resting state) [6], [7], [8], [9], [10] due to oxygen consumption and delivery. Therefore, novel, fast oxygen-sensing technologies are required for the investigation of these physiologic or pathophysiologic phenomena [11], [12].

Recently, a novel technology for fast oxygen sensing based on Multi Frequency Phase Fluorimetry (MFPF), termed NeoFox® (OceanOptics, Dunedin, USA; www.oceanoptics.com), was released. This technology is suitable for 10 Hz oxygen measurements using fluorescent dye sensors such as the FOXY-AL300 (OceanOptics, Dunedin, USA). This indwelling, uncoated ruthenium tipped fibre optic probe allows for rapid measurement of oxygen within blood or tissue. The technology, however, has not yet been compared to conventional polarographic Clark-type electrode (CTE)-based P_O2_ measurements [13], [14].

This study aimed to validate MFPF-P_O2_ measurements versus CTE-based measurements of oxygen partial pressure. Firstly, in a porcine lung model, MFPF-P_O2_ was measured *in vitro* without the interference of blood flow using two FOXY-AL300 probes (OceanOptics, Dunedin, USA) and was then compared to CTE blood gas P_O2_ analysis. Secondly, for further validation, we developed an artificial extracorporeal circulation setup primed with a mixture of crystalloid, colloid and human packed red cells to imitate *in vivo* conditions. In this setup, MFPF-P_O2_ readings were assessed and then compared to conventionally measured P_O2_ by CTE. Furthermore, in the artificial circulation setup, temperature and blood flow were randomly varied in order to evaluate their impact on MFPF-P_O2_ measurements. In addition, MFPF FOXY-AL300 (OceanOptics, Dunedin, USA) probe response times were determined in the gas- and blood-phases to further assess this novel technology. In summary, the overall aim could be achieved.

## Materials and Methods

### MFPF Technological Principle

The MFPF platform (NeoFox®, OceanOptics, Dundin, USA) was mounted with the indwelling FOXY-AL300 (OceanOptics, Dunedin, USA) aluminium-jacketed optical fibre probe (500 µm outer and 300 µm core diameter) with an uncoated ruthenium complex at the tip to assess luminescence lifetime in the region surrounding the fluorescent dye, which was roughly equal to the fibre-optic tip surface area (about 0.3 mm^2^). MFPF assesses luminescence lifetime by modulation of light excitation frequency where the oxygen dependent phase shift of the emission is detected. Lifetime is related to phase shift by the equation: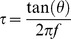, where τ is calculated lifetime in s, f is LED modulation frequency in Hz, and θ is phase shift measured by the instrument. The signals are transmitted via connected glass fibre cable to the MFPF server (NeoFox®, OceanOptics, Dundin, USA) with an integrated light emitting diode, as well as temperature and atmospheric pressure transducers. An external thermistor probe compensates for temperature changes (once a multi-point temperature calibration is performed). Lifetime data were logged by NeoFox® Software (OceanOptics, Dunedin, USA) for calculation of absolute P_O2_ values. Absolute P_O2_ values, luminescence phase shift, luminescence intensity, temperature and ambient pressure were displayed at a digital sampling rate of 10 Hz.

### Calibration of the FOXY-AL300 Oxygen Probe

P_O2_ probes were calibrated at different oxygen levels and temperatures in a closed thermostat controlled gas-tight chamber according to the manufacturer's instructions. The chamber was purged with pure nitrogen (P_O2_ = 0 mmHg), compressed air (P_O2_ = 158 mmHg) and pure oxygen (P_O2_ = 750 mmHg), respectively, in consideration of atmospheric pressure with a fixed laminar flow of 2 L min^−1^ at 20, 30 and 40°C. After stabilisation, the corresponding lifetimes were used to create a multi-temperature calibration table.

### Ethics statement

This study was carried out in strict accordance with the recommendations in the Guide for the Care and Use of Laboratory Animals of the National Institute of Health. The protocol was approved by the Animal State Care and Use Committee of the Rhineland Palatinate, Germany (Permit Number: G07-10-013). All surgery was performed under deep anaesthesia, and all efforts were made to minimise suffering [15]. Animal experiments were performed at the Department of Anesthesiology, Medical Center of the Johannes Gutenberg-University, Mainz, Germany and laboratory experiments at the Department of Anesthesiology and Pain Therapy, Inselspital, Bern University Hospital, and University of Bern, Switzerland.

### MFPF Validation *in vitro* using a porcine lung model

Eight piglets, *Sus scrofa domestica* (20±2 kg), were investigated. After the induction of anaesthesia with a bolus of fentanyl 5 µg kg^−1^ i.v., propofol 2 mg kg^−1^ i.v. and single shot atracurium 0.5 mg kg^−1^ i.v. via an ear vein, the pigs were orotreachally intubated (ID 8.0 mm cuffed tubing) and ventilated in pressure-controlled mode (AVEA, Viasis Healthcare, Höchberg, Germany). Vascular access was achieved by surgical cut down for the placement of an arterial blood pressure line in the femoral artery. Normocapnia (P_aCO2_ 35–45 mmHg) was maintained with a tidal volume of 6–8 ml kg^−1^ BW^−1^. Anaesthesia was maintained by continuous infusion of fentanyl (5 µg^−1^ kg^−1^ h^−1^ i.v.) and propofol (5–10 mg^−1^ kg^−1^ h^−1^ i.v.). During the experiment, F_IO2_ was adjusted (0.21, 0.4, 0.6, 0.8 and 1.0) to obtain P_O2_ readings over the whole normobaric measurement range. Core temperature by rectal probe was maintained within the physiologic range (37.0–38.5°C) using a heating blanket.

Blood samples (10 ml each) were withdrawn at each F_IO2_ level into heparinised gas tight syringes covered with opaque tape. For each sample, the tips of two FOXY-AL300 probes (OceanOptics, Dunedin, USA) were placed into the syringe outlet sealed with a rubber port to avoid air contact. After reaching steady state values, both MFPF-P_O2_ readings were simultaneously recorded for 2 min using the corresponding animal core temperature for the measurement. Then, the same blood sample was analysed by conventional polarographic Clark-type electrodes (RapidLab 415, BayerHealthcare, Leverkusen) immediately afterwards.

### MFPF validation *ex vivo* using an artificial circulatory setup

**Figure 1:**
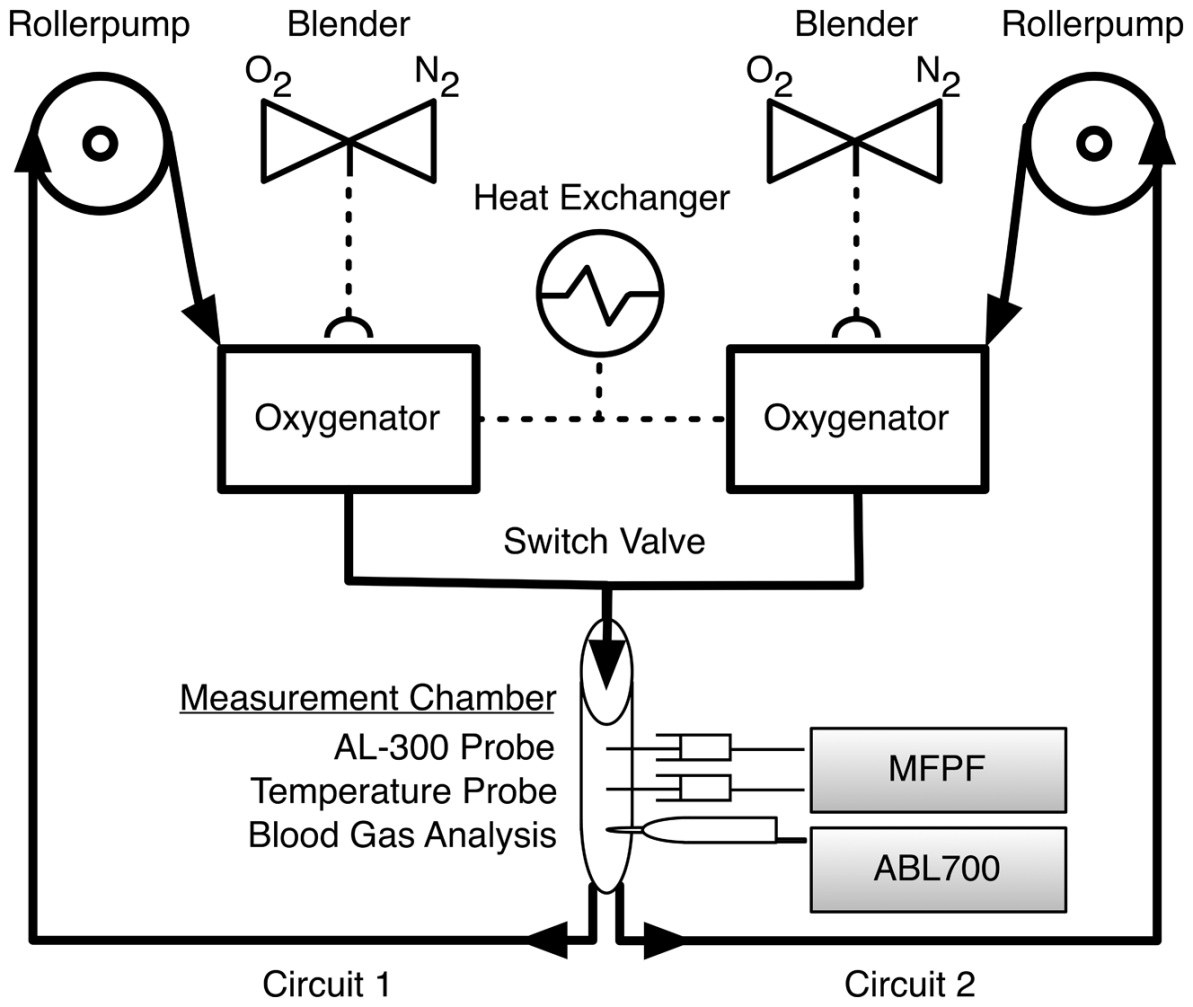
Artificial Circulatory Setup. Artificial circulatory setup with two independent circuits filled with human packed red blood cells (haematocrit of 30%). A switching valve between the circuits enabled a step-change between highly oxygenated (circuit 1: purged with pure oxygen) and oxygen free blood (circuit 2: purged with nitrogen). Black arrows represent direction of blood flow. Adapting the settings of the rollerpumps and the heating-cooling device (heat exchanger) allowed blood-flow and temperature to be controlled. Via the O_2_/N_2_ blenders, oxygen content could be adapted at fixed sweep gas flow over the oxygenators. Measurement chamber contained 1.) ports for insertion of MFPF probes (Foxy-AL 300); 2.) a temperature probe and 3.) a sampling port for Clark-typed based (CTE) P_O2_ analysis (ABL 700).

In order to provide standardised conditions and to simulate *in vivo* conditions, an artificial circulatory system was set up. The apparatus was built of: a) two oxygenators (Jostra Quadrox D, Maquet Cardiopulmonary AG, Hirrlingen, Germany), b) 3/8 inch silicon tubing (Fumedica, Muri, Switzerland) with 3/8–3/8 inch connecting pieces (Luerlock, Fumedica, Muri, Switzerland), c) two multi-flow roller pumps (Stöckert, Munich, Germany), d) a heat exchanger (HCU-30, Maquet Cardiopulmonary AG, Hirrlingen, Germany) and e) an ambient light protected measurement chamber (Figure 1). A switch valve between two independent closed circuits enabled the induction of a step change between highly oxygenated blood (F_IO2_ 1.0, circuit 1) to oxygen free blood (F_IO2_ 0.0, circuit 2) in order to assess the time constants of the FOXY-AL300 probes in blood-phase.

The artificial circulatory system was primed with a mixture of crystalloid, colloid and human packed red cells to achieve a haematocrit of 30% and electrolyte values within the physiological limits. This setup allowed for control and variation of blood flow (by variation of rpm at the rollerpumps), temperature (by variation at the membrane equilibrator) and oxygen content (by P_O2_ variation at the blender).

In order to get measurements over the normobaric measurement range for the validation of MFPF against Clark-type electrode P_O2_, F_IO2_ was set at random according to a computer generated list [16] from 0.0 to 1.0. To achieve an F_IO2_ of less than 0.21, a mixture of oxygen and nitrogen was used. For all measurements, duplicate MFPF P_O2_ readings (2 FOXY-AL300 probes) were recorded for 1 min duration alongside blood withdrawal for conventional polarographic Clark-type electrode P_O2_ analysis (ABL 700, Radiometer, Copenhagen, Denmark).

Measurements were performed according to the following protocols:

1. To assess the agreement of MFPF vs. Clark-type electrode P_O2_ at physiological body temperature conditions: at a temperature of 37°C, blood flow of 1.6 L min^−1^ and a sweep gas flow of 1 L min^−1^; 55 measurements were performed at varying P_O2_ levels.
2. To assess the impact of temperature: at a blood flow of 0.5 L min^−1^ and sweep gas flow 0.5 L min^−1^, temperature was set at random according to a computer generated list [16] to 20, 30 or 40°C. For each temperature level, 15 measurements were performed at varying P_O2_ levels.
3. To assess the impact of blood flow: at a temperature of 37°C and a sweep gas flow of 2 L min^−1^, blood flow was set at random according to a computer generated list [16] to 0.8, 1.6, 2.4, 3.2 and 4.0 L min^−1^. For each blood flow level, 10 measurements were performed at varying P_O2_ levels.

### Statistical Analysis

Descriptive and statistical data analysis was performed using SPSS® V.19 (IBM Inc., New York, USA). For metric variables, statistical measures such as mean and standard deviation (mean±SD) were calculated. Linear regression analysis upon P_O2_ values measured by MFPF and CTE were performed. Bias and precision values were calculated by the Bland-Altman method [17], [18]. The comparability was presented in explorative manner by description of mean differences, range of differences and 1.96-SD limits (lower and upper limit of agreement) [19]. In addition, linear regression analysis of difference versus mean was performed and then tested, if the slope is significantly different from zero. Furthermore, intra-class correlation between the two MFPF probes was assessed. To test the influence of blood flow and temperature variation, a multiple linear regression model was fitted: magnitude of P_O2_ measurements [(CTE+MFPF)/2], temperature and blood flow as independent variables; differences of MFPF and CTE as dependent variable. The categories of temperature and blood flow were handled with dummy coding. The analyses of data were done in explorative manner and the outcome of a statistical test with a *P*-value<0.05 was interpreted as statistically significant.

## Results

### MFPF Validation *in vitro* using a porcine lung model

**Figure 2:**
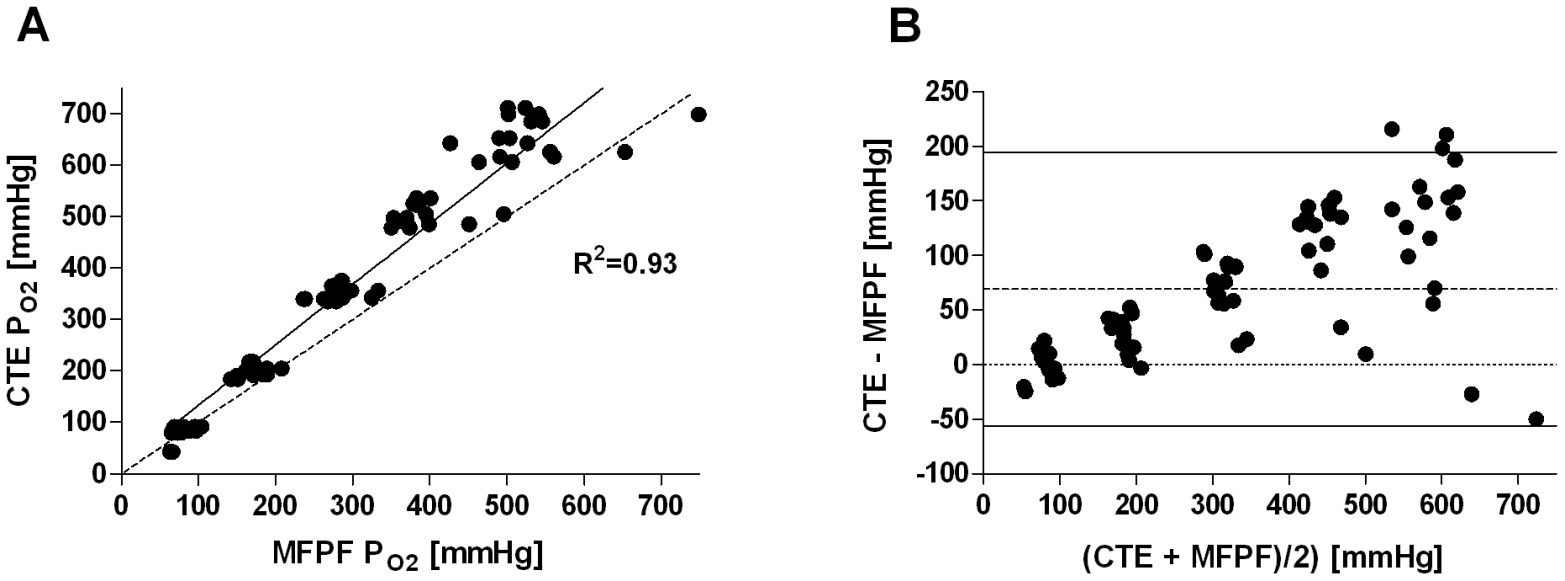
Multi Frequency Phase Fluorimetry P_O2_ vs. Clark-type Electrode P_O2_ (porcine blood *in vitro*, normobaric range). Panel A: Linear regression plot, the solid line displays the line of best fit, the dashed line shows the line of identity; Panel B: Bland-Altman plot showing the differences (CTE-MFPF) versus the means for absolute P_O2_ values. The dashed line represents the bias, the solid lines the 1.96 standard deviation interval.

Replicated porcine blood sampling revealed 80 duplicate MFPF-P_O2_ readings (range 63 to 749 mmHg) with the corresponding CTE samples (range 43 to 712 mmHg). The linear regression was described by the equation CTE = 15.59+1.18*MFPF (R^2^ = 0.93; *P*<0.0001) (Figure 2A). Agreement by Bland Altman analysis showed a mean difference (mean_diff_) in P_O2_ between CTE and MFPF of 69.2±64.1 mmHg (Figure 2B). The differences ranged from −50.1 to 215.6 mmHg within the 1.96-SD limits (lower limit -56.3, upper limit 194.8 mmHg). Linear regression of the difference versus mean showed CTE-MFPF = −0.64+0.20*[(CTE+MFPF)/2] (R^2^ = 0.36, slope significantly different from zero: *P*<0.0001). Intra-class correlation between the two MFPF probes showed a reproducibility of R^2^ = 0.98.

### MFPF validation *ex vivo* in artificial circulatory setup

**Figure 3:**
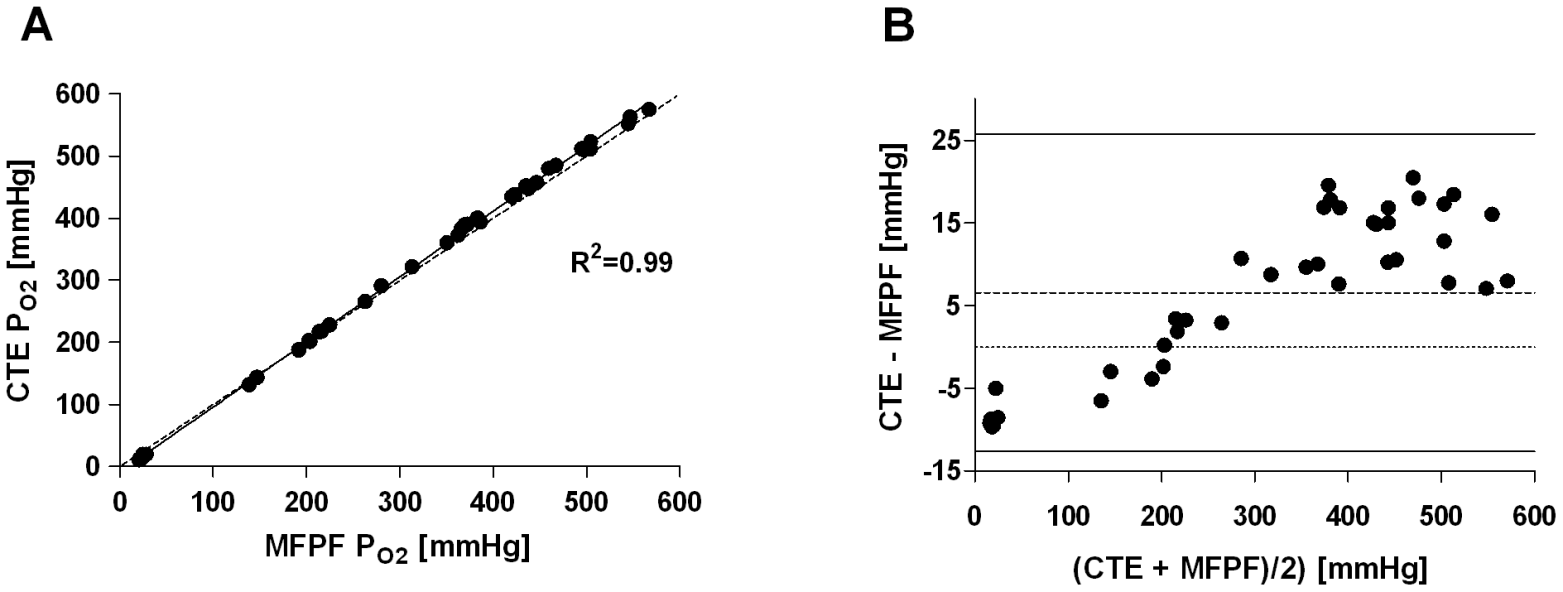
Multi Frequency Phase Fluorimetry P_O2_ vs. Clark-type Electrode P_O2_ (human blood *ex vivo*, normobaric range). Panel A: Linear regression plot, the solid line displays the line of best fit, the dashed line shows the line of identity; Panel B: Bland-Altman plot showing the differences (CTE-MFPF) versus the means for absolute P_O2_ values. The dashed line represents the bias, the solid lines the 1.96 standard deviation interval.

**Figure 4:**
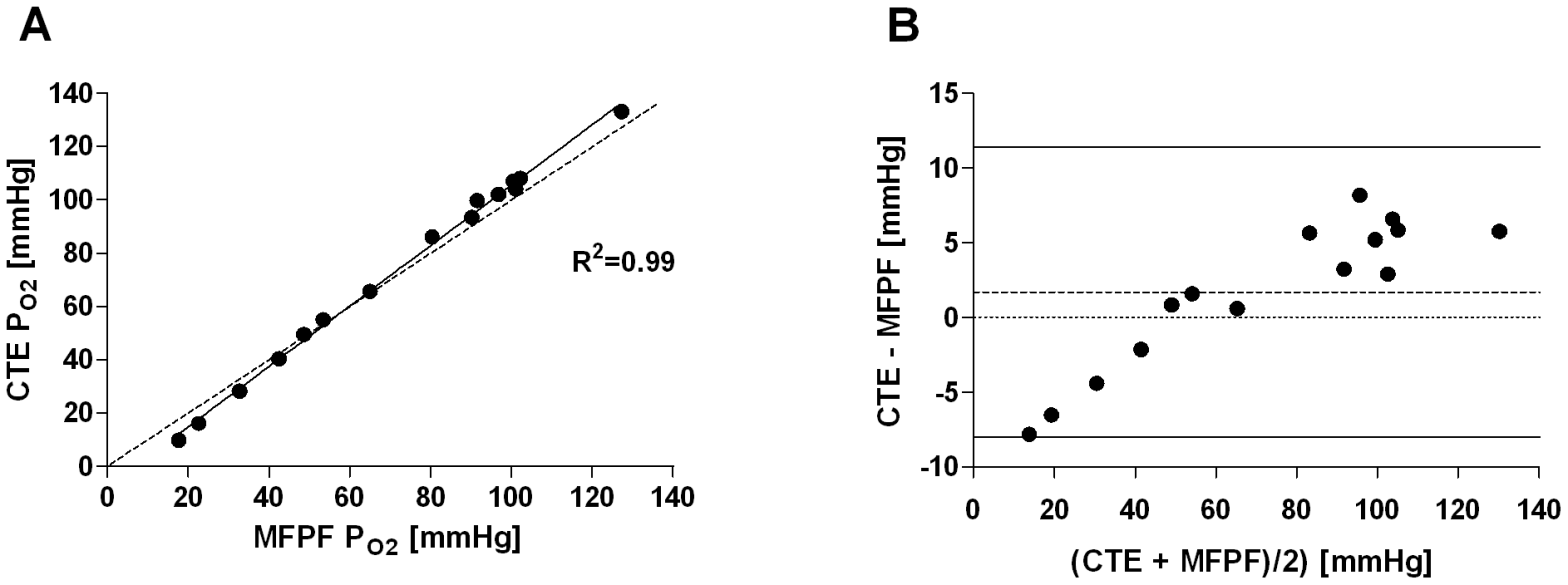
Multi Frequency Phase Fluorimetry P_O2_ vs. Clark-type Electrode P_O2_ (human blood *ex vivo*, hypoxic and normoxic range). Panel A: Linear regression plot, the solid line displays the line of best fit, the dashed line shows the line of identity; Panel B: Bland-Altman plot showing the differences (CTE-MFPF) versus the means for absolute P_O2_ values. The dashed line represents the bias, the solid lines the 1.96 standard deviation interval.

In artificial circulatory setup, MFPF-P_O2_ measurements ranged from 20 to 567 mmHg and the corresponding CTE samples from 11 to 575 mmHg. Linear regression yielded the equation: CTE = −8.73+1.05*MFPF (R^2^ = 0.99; *P*<0.0001) (Figure 3A). Bland-Altman analysis calculated a mean difference (mean_diff_) between CTE and MFPF P_O2_ measurements of 6.6±9.8 mmHg. Differences ranged from −9.7 to 20.5 mmHg within the 1.96-SD limits (lower limit -12.7, upper limit 25.8 mmHg) (Figure 3B). Linear regression of the difference versus mean showed CTE-MFPF = −8.62+0.05*[(CTE+MFPF)/2] (R^2^ = 0.78, slope significantly different from zero: *P*<0.0001). Linear regression in a sub-set of P_O2_ between 0 and 140 mmHg yielded CTE = −7.84+1.13*MFPF (R^2^ = 0.99; *P*<0.0001) (Figure 4A). Bland-Altman analysis of this sub-analysis showed a mean difference of 1.7±4.9 mmHg (Figure 4B). The differences ranged from −7.8 to 8.1 mmHg within the 1.96-SD limits (lower limit -8.0, upper limit 11.4 mmHg). Linear regression of the difference versus mean yielded CTE-MFPF = −7.47+0.13*[(CTE+MFPF)/2] (R^2^ = 0.84, slope significantly different from zero: *P*<0.0001). In total, reproducibility of R^2^ = 0.99 between the two MFPF probes (intra-class correlation) was depicted.

### Impact of temperature and blood flow on MFPF P_O2_ measurements

The influence of the temperature upon the differences of CTE and MFPF P_O2_ measurements reached from −0.6 mmHg (temperature = 20°C) to 31.8 mmHg (temperature = 40°C) (Table 1). The influence of blood flow on difference in P_O2_ of the two methods reached from 0.6 mmHg (blood flow = 1.6 L min^−1^) to 14.8 mmHg (blood flow = 0.8 L min^−1^) (Table 1). The linear regression model with the magnitude of P_O2_ measurements [(CTE+MFPF)/2], temperature and blood flow as independent variables showed a significant influence upon the magnitude of P_O2_ measurements and a non-significant influence of temperature or blood flow upon the differences of P_O2_ measurements (dependent variable) (Table 2).

**Table 1:** Influence of temperature and blood flow on MFPF P_O2_ measurements.

	P_O2_ range [mmHg]	N	MFPF-CTE mean	MFPF-CTE SD	MFPF-CTE median
**Temperature [**°**C]**					
20.0	0–140	5	−0.6	4.6	0.9
30.0	0–140	5	0.4	5.9	0.6
40.0	0–140	5	5.3	2.4	5.8
**Total**		**15**	**1.7**	**5**	**2.9**
20.0	140–600	12	13.3	28.7	28.6
30.0	140–600	11	29.4	34.9	35.9
40.0	140–600	11	31.8	21.9	40.1
**Total**		**34**	**24.5**	**29.4**	**29.3**
**Flow [L min^−1^]**					
0.80	0–600	9	14.8	4.5	17.3
1.60	0–600	7	.6	9.7	−2.4
2.40	0–600	8	5.4	10.5	8.6
3.20	0–600	7	6.5	10.1	10.1
4.00	0–600	9	4.1	9.4	7.1
**Total**		**40**	**6.6**	**9.8**	**8.4**

P_O2_  =  oxygen partial pressure [mmHg]; MFPF  =  Multi Frequency Phase Fluorimetry; CTE  =  Clark-type electrode; SD  =  standard deviation.

**Table 2:** Influence of temperature and blood flow on MFPF P_O2_ measurements: Linear regression model.

	Non-standardized coefficients			95.0% CI	
Independent variable	Regression coefficient	Standard error	*P*-value	lower limit	upper limit
**Temperature (base 20 °C)**					
Constant	−2.335	10.483	0.825	−23.744	19.074
P_O2_-magnitude [(MFPF + CTE)/2]	0.062	0.027	0.031	0.006	0.118
30°C	12.854	11.466	0.271	−10.563	36.271
40°C	19.635	11.392	0.095	−3.629	42.9
**Blood flow (base 0.8 L min^−1^)**					
Constant	−4.707	2.401	0.058	−9.586	0.171
P_O2_-magnitude [(MFPF + CTE)/2]	0.045	0.004	<0.001	0.037	0.054
1.6 L min^−1^	−4.461	2.441	0.076	−9.422	0.5
2.4 L min^−1^	−2.822	2.262	0.221	−7.42	1.776
3.2 L min^−1^	−2.093	2.328	0.375	−6.825	2.638
4.0 L min^−1^	−5.188	2.171	0.023	−9.601	−0.776

Linear regression model: magnitude of P_O2_ measurements, temperature and blood flow as independent variables, differences of P_O2_ measurements as dependent variable. P_O2_  =  oxygen partial pressure; MFPF  =  Multi Frequency Phase Fluorimetry; CTE  =  Clark-type electrode; CI  =  confidence interval.

### MFPF FOXY-AL300 Response Time

**Figure 5:**
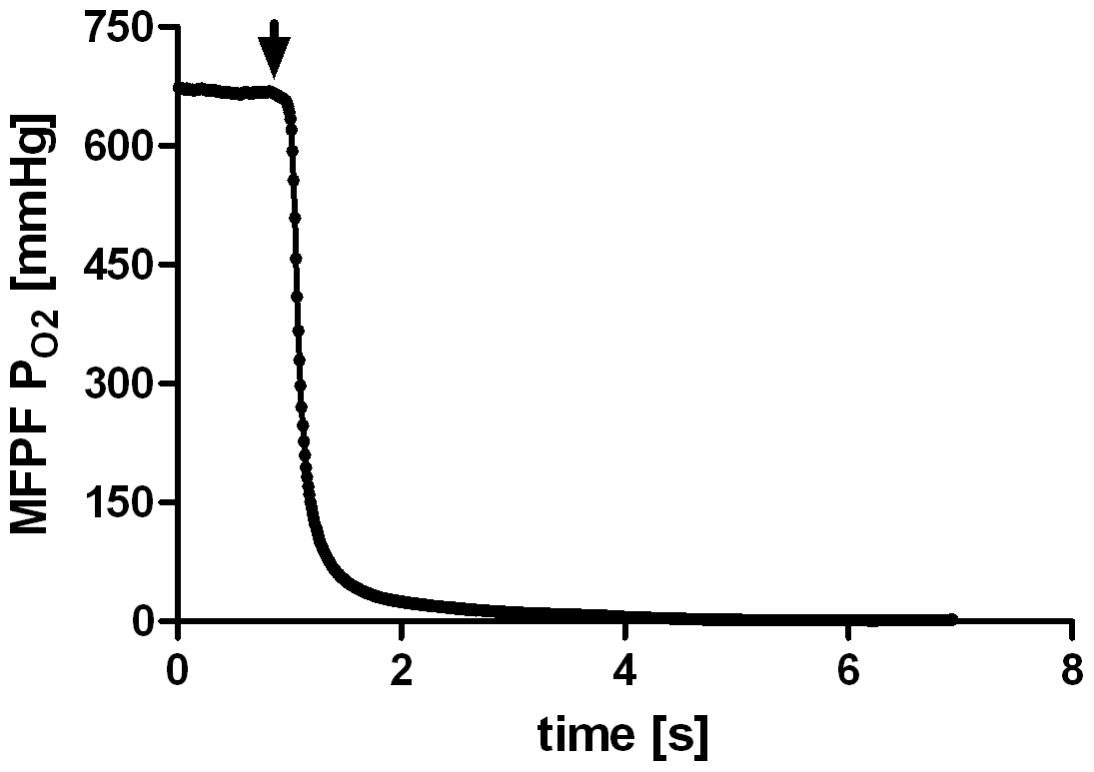
Multi Frequency Phase Fluorimetry/FOXY-AL300 Probe Response Time. Example of an MFPF step-down manoeuvre in artificial circulatory setup (human blood-phase). The graph displays the absolute MFPF P_O2_ values over the time course. The arrow marks the time when the switching valve was changed between the oxygenated (750 mmHg) and non-oxygenated blood (0 mmHg) circuit.

MFPF in combination with the uncoated FOXY-AL300 probes showed a mean response time of 1.48±0.26 s in gas-phase and 1.51±0.20 s in blood-phase after a step-change from pure oxygen (P_O2_ = 749 mmHg) to pure nitrogen (P_O2_ = 0 mmHg) at a digital sampling rate of 10 Hz (Figure 5). The monoexponential response time by Boltzmann fitting represents the time it takes the system to reach 63.2% of its final asymptotic value.

## Discussion

Findings demonstrate MFPF technologýs capacity for detecting changes in P_O2_ compared to Clark-type electrode (CTE) based P_O2_ measurements. For discrete samples, *in vitro* porcine blood phase measurements (R^2^ = 0.93) did not give very tight limits of agreement. However, this study was not designed to validate MFPF as replacement for traditional *in vitro* blood gas analysis. For such a setup, we would have needed a configuration where the sensor and sample are part of a heated block with very tight control of both probe and blood sample temperature. The authors want to point out that the real application of MFPF is for flowing blood when the probe is placed *in vivo* - which was investigated by *ex vivo* measurements in an artificial circulatory setup filled with human blood. These findings demonstrate that MFPF technology provides high accuracy for P_O2_ measurements compared to CTE based P_O2_ measurements (R^2^ = 0.99). The *ex vivo* measurements showed that MFPF technology accurately compensates for variation in temperature and that P_O2_ measurements are not affected by variation in blood flow. MFPF response time in combination with the indwelling FOXY-AL300 probes was 1.48±0.26 s in the gas-phase and 1.51±0.20 s in the blood-phase (step-change from pure oxygen to pure nitrogen).

In summary, MFPF technology allows for fast, accurate and valid P_O2_ readings over the normobaric P_O2_ range (0–749 mmHg) for *ex vivo* experiments, as MFPF P_O2_ measurements ranged between 11 and 749 mmHg. This fact is the most important, as novel oxygen sensing technologies should be validated over the whole measurement range.

The *in vitro* animal model (porcine blood) showed good correlation (R^2^ = 0.93), although the data was determined in clusters of oxygen concentration (F_IO2_ 0.21, 0.4, 0.6, 0.8, 1.0). The observed high bias with positive drift as well as the enlarged standard deviation can be most likely explained by the systemic experimental error to use the animals' core temperature for the *in vitro* measurements. The main intention of this first series was to obtain readings without the influence of blood flow. Therefore, we focused on how to avoid air contact to the withdrawn blood and decided not to insert the temperature probe into the syringes. However, we did not expect such an impact of temperature, which presumably seems even more evident at high P_O2_-levels (Figure 2B). This finding can be explained by the underlying MFPF technology, as in fluorescent quenching, signal intensity increases at lower P_O2_ values. To fully understand the observed discrepancy of the *in vitro* (porcine blood) and *ex vivo* (human blood) results, we evaluated the difference of animal core temperature and blood temperature in the syringes two minutes after withdrawal post hoc in one further animal and found an error range of 0.9 to 1.6°C in ten repeated measures. Nevertheless, in the interesting physiologically hypoxic and normoxic (0–140 mmHg) P_O2_ range, porcine results show that MFPF technology provides good agreement (Figure 2B) for discrete samples, although in our setup the technique was less accurate for high P_O2_ values. Due to the underlying technological principle precision of MFPF, P_O2_ values should increase within this physiologically interesting range (0–140 mmHg), which is supported by the present data. Thus, it seems unlikely that MFPF technology characteristics were responsible for this discrepancy, as intra-class correlation between the MFPF probes was extremely high (R^2^ = 0.99) in porcine setup and MFPF measurements in the artificial circulatory setup (human blood) showed excellent agreement to CTE, even at high P_O2_ levels. From our point of view, the porcine results nicely demonstrate the high temperature sensitivity of MFPF measurements and further highlight the importance of correct temperature acquisition. Instead of repeating this series, we decided to develop a new artificial circulatory system for further validation, where we could also investigate the influence of temperature and blood flow by standardised variation of these physiological values.

Under *ex vivo* conditions in the artificial circulatory setup (human blood), we found excellent correlation (R^2^ = 0.99) of MFPF and CTE P_O2_ with measurements equally distributed over the entire normobaric range (Figure 3). In general, MFPF values were slightly lower in comparison to CTE values. These findings were unexpected, as the MFPF technology, unlike polarography, does not consume any oxygen. These results might be explained by the fact that the blood-gas-analysers used allow a potential bias of 5% in calibration drift and it is well known that these are not really linear over the entire range. This underestimation decreases with lowered P_O2_ values and is therefore of limited clinical importance (Figure 2, 3).

In general and as demonstrated by porcine results, temperature is obviously one major confounder on P_O2_ measurement [20], [21]. However, the present results of the artificial circulatory setup show that the MFPF technology accurately compensates for alterations in temperature if assessed strictly at the measurement site. This fact highlights the efficiency of multi-temperature compensation performed by MFPF software between 20 and 40°C. In this context, it would be desirable to have sensors available with an integrated temperature thermistor to prevent errors such as temperature probe misplacement. In addition to temperature, blood flow may influence P_O2_ measurements of an indwelling sensor [22]. The present *ex vivo* results confirm that MFPF technology correctly measures P_O2_, even under variations of blood flow within the range of 0.8 to 4.0 L min^−1^.

One major advantage of MFPF P_O2_ readings, next to high agreement and reproducibility, is high temporal resolution. We measured the monoexponential time constant for a step change in P_O2_ at 1.5 s in blood. In contrast, the polagraphic Licox probe showed response times up to several min [5], [23]. Recently, faster P_O2_ probes, such as the Neurovent PTO probe based on the fluorescent quenching of oxygen, have been presented. Although these probes reported higher response times (30 – 120 s), none of the commercially available technologies come near the temporal resolution of the uncoated ruthenium probe used in the current study [23], [24], [25], [26], [27], [28].

The MFPF system is easy to set up and needs no complex calibration. In this validation study, the FOXY-AL300 probes were individually calibrated to minimise for potential errors, but in less demanding use, a calibration file supplied by the manufacturer for each probe can be used with acceptable accuracy. Furthermore, phase shift is not influenced by the medium the oxygen sensor is placed in, allowing simplified calibration in gas phase. Moreover, phase shift measurement is not influenced by the probe indicator dye concentration, photo bleaching of dye, or excitation source intensity. This results in stable signals with high signal to noise ratio [29].

Unfortunately, we could not obtain readings in a clinical setting in patients, as the indwelling probes available up to now (e.g. the FOXY AL-300) are not licensed for human application. Therefore, we investigated the MFPF technology *ex vivo* in a self-built artificial circulatory system filled with human blood. Although ruthenium-based dyes are commonly used in biological monitoring applications and are thought to be non-toxic, for future application in humans, the metallic surface of the probe and its tip containing ruthenium need to be coated with silicone or Teflon®. In contrast to a silicone probe coating, Teflon® would allow for more rapid diffusion of oxygen and thereby maintain ultrafast probe response time. The challenge for the sensor engineering community will be to avoid direct contact of the fluorescent dye to blood components. Also, it is necessary to avoid protein absorption and blood clotting to maintain signal stability and accuracy. Therefore, an intensive research effort is still necessary, but if engineering is to advance from laboratory-based research to commercial production levels, the MFPF technique could provide clinicians with an effective tool.

A limitation of the study is that we did not explicitly investigate the measurement stability over time. However, the present findings showed no calibration drift of the probes.

In summary we conclude that *ex vivo* MFPF-P_O2_ readings are reproducible, and show excellent correlation and high agreement with the gold standard Clark-type electrode (CTE)-based P_O2_ analysis. This novel technology adequately compensates for changes in temperature and allows for accurate measurements at various blood flow states. This feasible, accurate and easy to calibrate method has the potential to dynamically follow changes in blood oxygenation under various physiologic and pathophysiologic conditions with a high temporal resolution.
